# Supplementary material for: Obtaining N-Enriched Mesoporous Carbon-Based by Means of Gamma Radiation
Source: Nanomaterials (Basel). 2022 Sep 12;12(18):3156. doi: 10.3390/nano12183156 (PMC9505784; doi:10.3390/nano12183156)
Supplement: Supplementary file 1 [file nanomaterials-12-03156-s001.zip › nanomaterials-1887386-supplementary materials.pdf]

nanomaterials

Article

Obtaining N-enriched mesoporous carbon-based by means of gamma radiation

Piotr Kamedulski [1,2,*], Malgorzata Skorupska [1], Izabela Koter [1], Maciej Lewandowski [1], Víctor Karim Abdelkader-Fernández [3] and Jerzy P. Lukaszewicz [1,2]

[1] Faculty of Chemistry, Nicolaus Copernicus University, Gagarina 7, 87-100 Torun, Poland
[2] Centre for Modern Interdisciplinary Technologies, Nicolaus Copernicus University, Wilenska 4, 87-100 Torun, Poland
[3] Department of Inorganic Chemistry, University of Granada, 18071 Granada, Spain
* Correspondence: pkamedulski@umk.pl

Section: Results in experiments

The morphology of the surface of the raw carbon materials was analysed using a high-resolution transmission electron microscopy (HRTEM, FEI Europe production, model Tecnai F20 X-Twin, Brno, Czech Republic). Representative images of the raw carbon materials are collected in Figure S1. HRTEM images presented in Figure S1 confirm the typical an amorphous structure of mesoporous activated carbons and the proposed method did not destroy and change the original morphology. In turn Figure S2–S4 show XPS spectra of investigated samples.

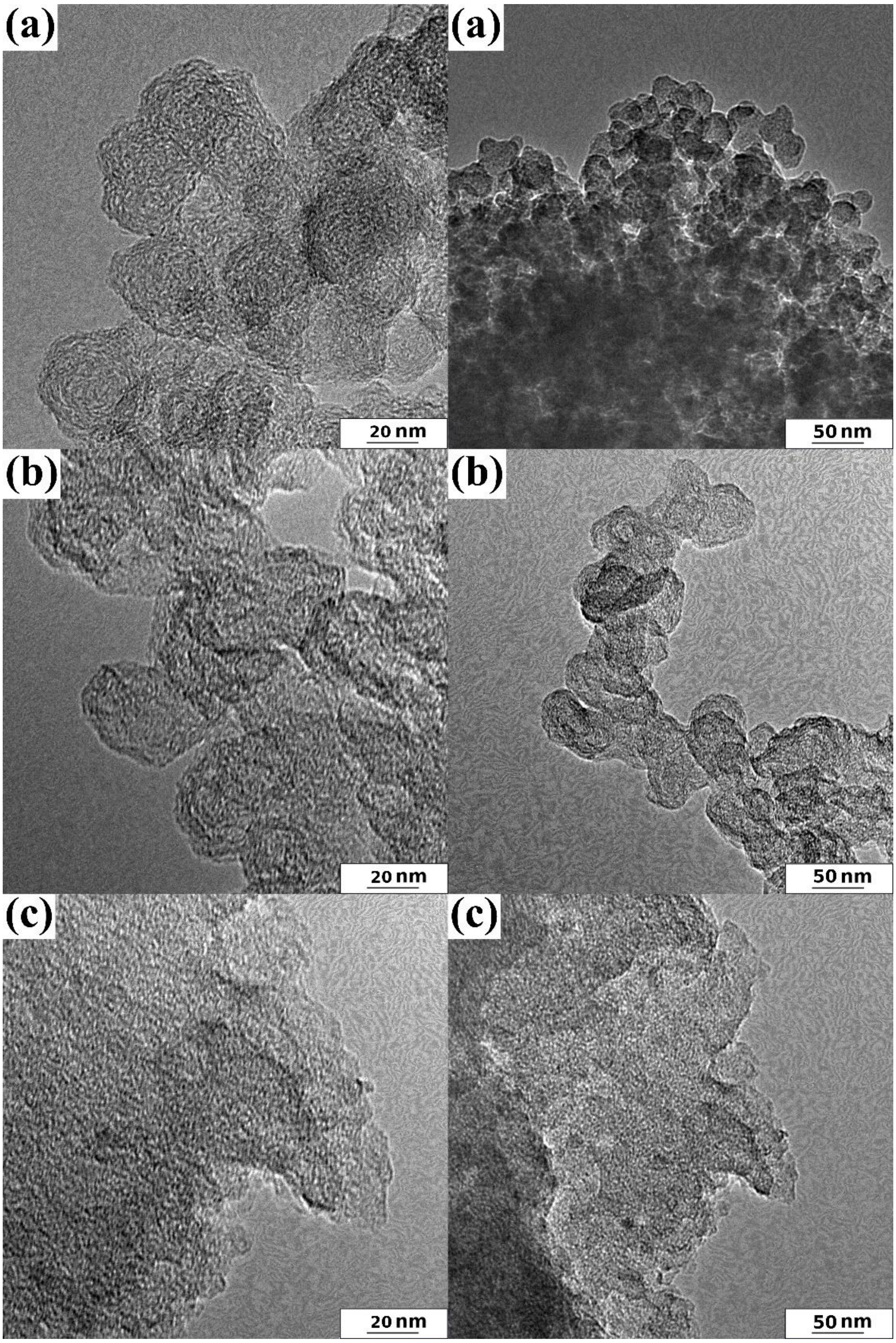

Figure S1. TEM images of raw carbons sample in different magnifications (**a**) AC-O, (**b**) AC-KB and (**c**) AC-N.

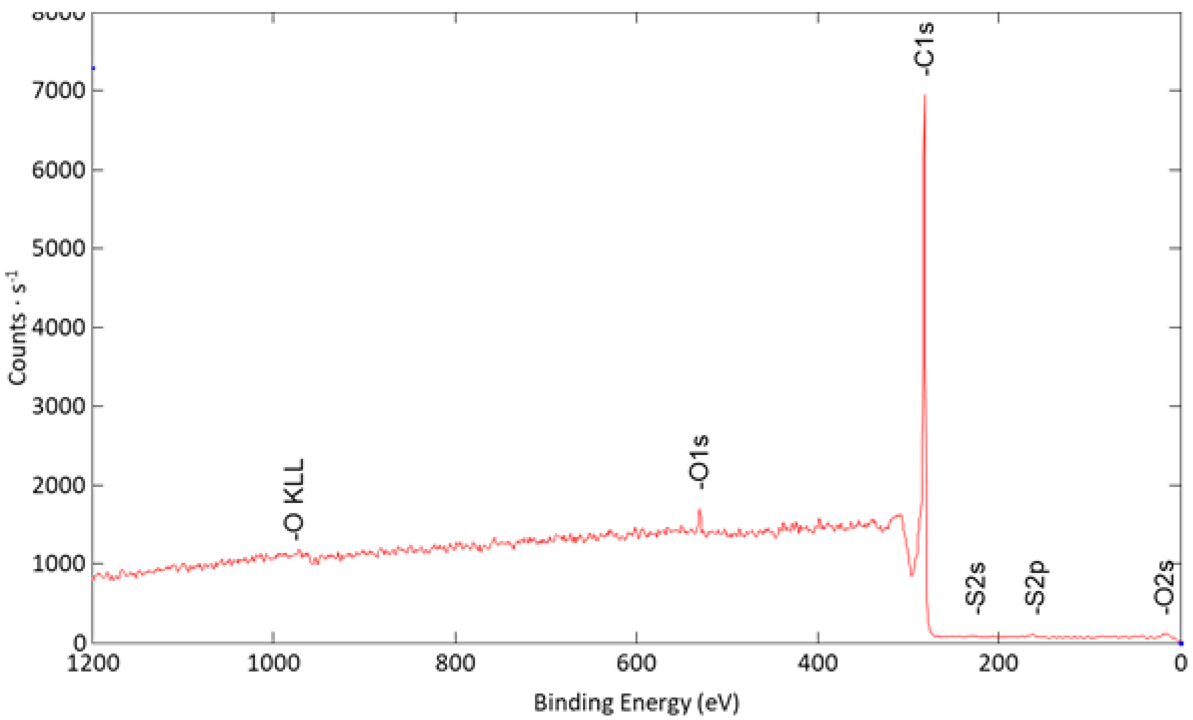

Figure S2. XPS image of AC-O-II sample.

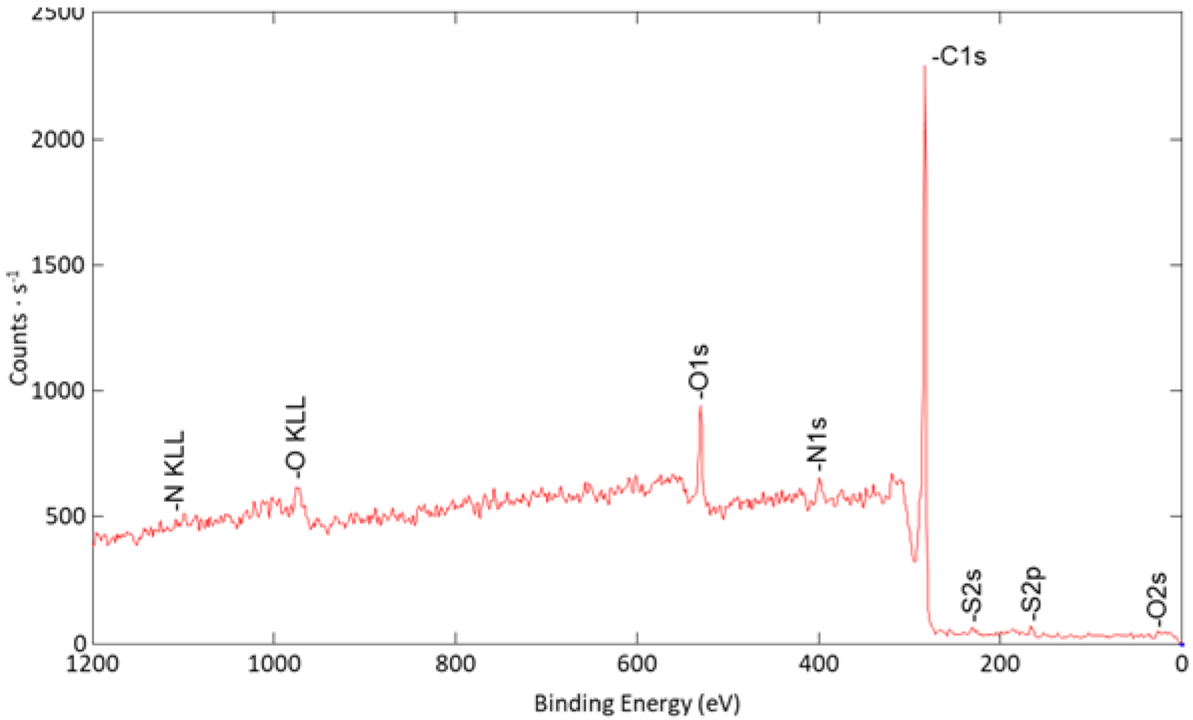

Figure S3. XPS image of AC-KB-II sample.

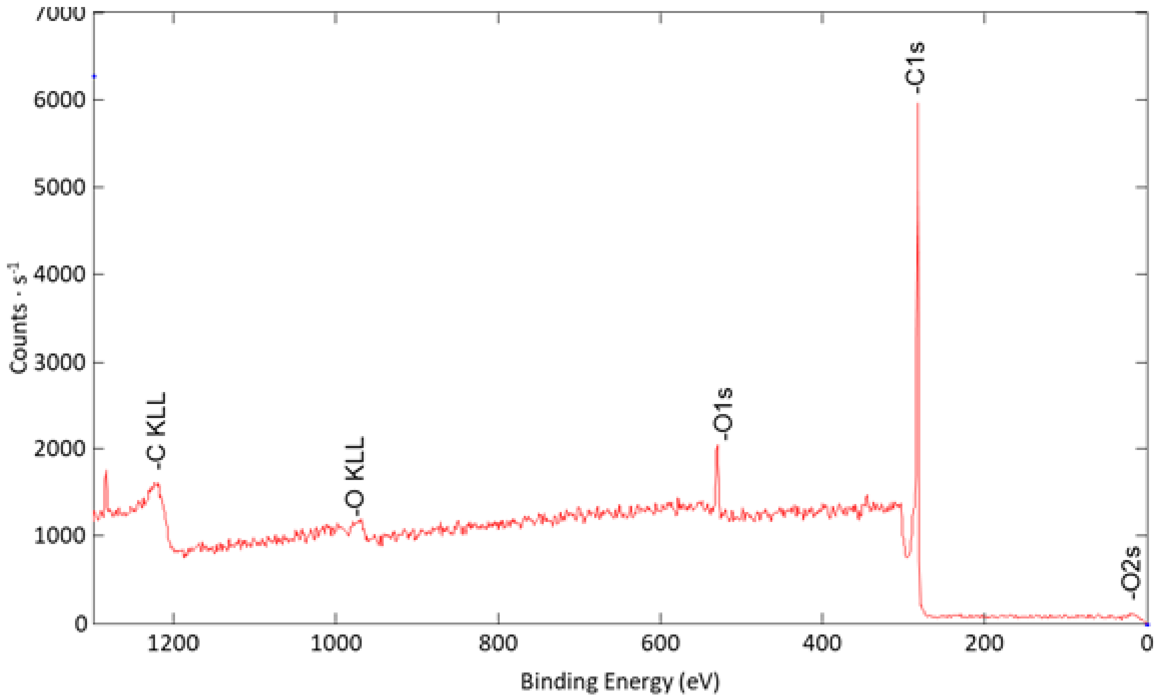

Figure S4. XPS image of AC-N-II sample**.**
